# Synergistic Effects of IMP‐1700, Ciprofloxacin, and X‐Ray Radiation in Bacteria and Mammalian Cell Lines: Implications for Use in Antimicrobial‐Resistant Bacteria

**DOI:** 10.1002/mbo3.70270

**Published:** 2026-04-27

**Authors:** Ida Vang Andersen, Ane Beth Sloth, Emilie Caroline Skuladottir Bøgestad, Christian Bernard Matthijs Poulie, Anne Skovsbo Clausen, Umberto Maria Battisti, Matthias M. Herth, Andreas Kjaer

**Affiliations:** ^1^ Department of Clinical Physiology and Nuclear Medicine & Cluster for Molecular Imaging, Copenhagen University Hospital – Rigshospitalet & Department of Biomedical Sciences University of Copenhagen Copenhagen Denmark; ^2^ Department of Drug Design and Pharmacology, Faculty of Health and Medical Sciences University of Copenhagen Copenhagen Denmark; ^3^ Department of Clinical Physiology, Nuclear Medicine & PET, Copenhagen University Hospital Rigshospitalet Copenhagen Denmark

**Keywords:** B16.F10, ciprofloxacin, HepG2, IMP‐1700, MDA.MB.231, NIH‐3T3, S. aureus, X‐ray

## Abstract

IMP‐1700 was developed as a compound designed to target bacterial DNA repair‐associated pathways, including homologous recombination mediated by the AddAB/RecBCD system. While its antibacterial properties are well established, the potential effects on mammalian noncancerous and tumor cells under genotoxic stress remain to be elucidated. This study investigates the impact of IMP‐1700 and its synergistic effect in combination with ciprofloxacin (CPX) and X‐ray radiation in bacterial cultures and mammalian cell lines. In *Staphylococcus aureus (S. aureus)*, neither IMP‐1700 (5 µM) nor CPX (15 µM) as monotherapy significantly reduced bacterial growth; their combination produced a substantial reduction in bacterial counts, and IMP‐1700 further enhanced killing when combined with 10 Gy X‐ray irradiation, resulting in a 77% decrease, demonstrating synergistic antibacterial activity. In mammalian cells, X‐ray cytotoxicity was cell‐type dependent: noncancerous NIH‐3T3 fibroblasts showed no significant effect, while B16.F10 melanoma cells displayed delayed sensitivity, MDA‐MB‐231 breast cancer cells an intermediate response, and HepG2 hepatocellular carcinoma cells marked radioresistance. IMP‐1700 enhanced radiation‐induced cytotoxicity across all cell lines, particularly at 72 h post‐irradiation. These findings suggest that although IMP‐1700 was designed to target bacterial DNA repair pathways, it can enhance sensitivity to genotoxic stress in both bacterial and mammalian systems. Further mechanistic investigation and safety evaluation are required before potential therapeutic application.

## Graphical abstract

IMP‐1700 enhances the cytotoxic effect on bacteria in combination with either ciprofloxacin (CPX) or X‐ray. In noncancerous and tumor cells, combination treatments markedly reduce viability with radiosensitizing effects. Further studies are needed to clarify the mechanism underlying IMP‐1700s effects on mammalian cells. Created in BioRender. Kjaer, A. (2025) https://BioRender.com/720o3eh

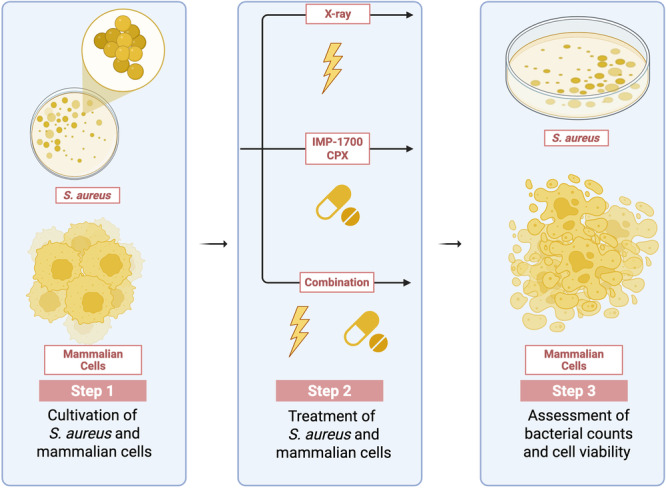

## 1 Introduction

The rise of antimicrobial resistance is a significant threat to modern medicine, limiting treatment options and increasing mortality worldwide (Naghavi et al. 2024). The widespread overuse and misuse of antibiotics, combined with the stagnation in novel drug development, have contributed to this crisis (Renwick et al. 2016). The financial loss of interest associated with antibiotics, largely due to the emergence of resistance, has led to a marked decline in the approval of new antimicrobial agents over the past few decades (Kinch et al. 2014). Consequently, there is an unmet clinical need for alternative therapeutic strategies that target essential bacterial processes beyond conventional antibiotic mechanisms (Lim et al. 2019), thereby circumventing resistance mechanisms.

One such approach involves targeting bacterial DNA repair mechanisms, which are crucial for survival under genotoxic stress (Brown and Wright 2016). The host's innate immune response naturally induces double‐stranded breaks (DSBs) in bacterial DNA during infections, and if unrepaired, these lesions are lethal to the bacteria (Lopez Chiloeches et al. 2021; Rigby and DeLeo 2012; Wigley 2013). Homologous recombination is a highly conserved DNA repair mechanism mediated by the AddAB/RecBCD enzyme complex (Ayora et al. 2011; Wigley 2013). These complexes recognize and process double‐stranded DNA breaks, degrading the DNA until encountering a specific Chi site. At this point, they generate a 3’ single‐stranded overhang suitable for RecA‐mediated homologous recombination and repair (Ayora et al. 2011; Yeeles and Dillingham 2010). In mammalian cells, DSBs are primarily sensed and processed by the MRN complex (MRE11‐RAD50‐NBS1) or by the Ku70/80 complex, which coordinate repair through homologous recombination (RAD51‐mediated) or non‐homologous end joining (NHEJ) (Ciccia and Elledge 2010). Unlike AddAB/RecBCD, mammalian complexes do not recognize Chi sites, and the mechanisms of strand resection and recombinase loading are distinct.

Quinolone antibiotics, such as ciprofloxacin (CPX), indirectly induce DSBs by inhibiting bacterial DNA gyrase and topoisomerase IV, essential enzymes responsible for maintaining DNA supercoiling and facilitating chromosome segregation during replication (Aldred et al. 2014; Beberok et al. 2018; Drlica et al. 1997). The growing prevalence of quinolone resistance has underscored the need for novel strategies to restore or enhance their efficacy. Small‐molecule inhibitors targeting bacterial DNA repair‐associated mechanisms have been proposed as potential adjuvants. One such compound is IMP‐1700, which was previously identified as capable of enhancing CPX activity against *Staphylococcus aureus* (*S. aureus*), though it modulates bacterial DNA damage responses (Lim et al. 2019).

An approach to the treatment of bacterial infection involves the use of X‐rays or internal radiation therapy (B. van Dijk, Allen, et al. 2020; B. van Dijk, Lemans, et al. 2020; Y. Zhang et al. 2023), a concept already established in oncology (Helal and Dadachova 2018; Larson et al. 2015). Both external and internal radiation therapies have shown potential in the treatment of bacterial infections (B. van Dijk, Lemans, et al. 2020). Conceptually, the ability of radiation to reach infected tissue and selectively target bacterial cells could be enhanced by conjugating radioactive isotopes to small molecules, such as IMP‐1700, thereby improving the precision and potency of the delivered radiation dose; this approach was not pursued in the present study.

**Figure 1:**
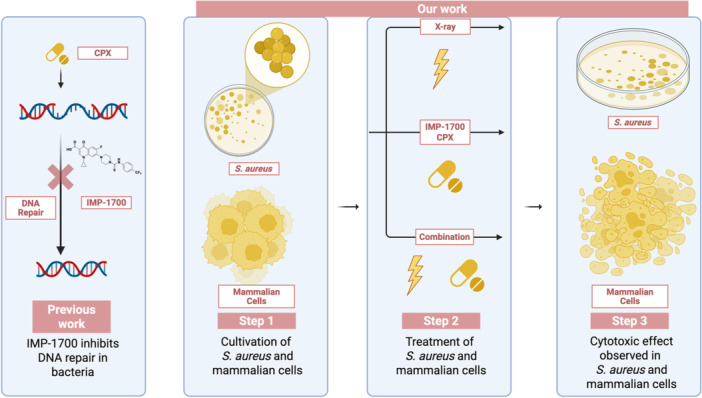
Graphical illustration of previous work by Lim et al. (2019) and the current experimental design investigating the effects of IMP‐1700 on bacterial and mammalian cells. Our work involved three different *S. aureus* strains, one noncarcinogenic mammalian cell line, and three different tumor cell lines. IMP‐1700 reduced bacterial viability in combination with ciprofloxacin (CPX) or X‐ray irradiation. In mammalian cells, IMP‐1700 decreased viability, with a reduction when combined with X‐rays. These results highlight the dual biological effects of IMP‐1700‐antibacterial activity and mammalian cytotoxicity and emphasize the need for further mechanistic studies to evaluate its therapeutic selectivity. Created in BioRender. Kjaer, A. (2025) https://BioRender.com/s1u5246.

In this study, we investigated the antibacterial efficacy and mammalian cytotoxicity of IMP‐1700 in three *S. aureus* strains (ATCC 29213, MH‐1670, and NCTC 8325), a noncancerous cell line NIH‐3T3, and three cancer cell lines (B16.F10, MDA‐MB‐231, and HepG2) (Figure 1).

To assess the safety profile of IMP‐1700, we examined its effects on noncancerous cells and cancerous mammalian cell lines under genotoxic stress. Specifically, we aimed to determine whether IMP‐1700 could selectively enhance bacterial killing while limiting cytotoxic effects in mammalian cells.

Our results demonstrate that in *S. aureus* ATCC 29213 bacteria, IMP‐1700 in combination with X‐ray irradiation reduced bacterial viability by 77%. In mammalian cells, viability was reduced by 83% in noncancerous NIH‐3T3 cells, and by 88%, 81%, and 100% in tumor cell lines B16.F10, MDA‐MB‐231, and HepG2, respectively, at 72 h post‐treatment. These findings suggest that IMP‐1700 in combination with X‐ray irradiation reduces bacterial viability, although the underlying mechanisms were not directly assessed. Its current lack of selectivity for bacterial over mammalian cells may limit its clinical applicability without further structural optimization or targeted delivery strategies.

## 2 Materials and Methods

### 2.1 Synthesis of IMP‐1700

All reactions involving dry solvents or sensitive agents were performed under an inert atmosphere, and glassware was dried before use. Commercially available chemicals were used without further purification. All solvents were High‐Performance Liquid Chromatography (HPLC) grade. Solvents were dried before use with an SG water solvent purification system or by standard procedures, and reactions were monitored by HPLC‐MS. Compounds were dried under high vacuum. ^1^H NMR spectra were recorded on a 600 MHz Bruker Avance III HD, and ^13^C NMR spectra on a 151 MHz Bruker Avance III HD. Data are reported in the following order: chemical shift (δ) [multiplicity (br, broad; s, singlet; d, doublet; dd, doublet of doublets; dt, doublet of triplets; t, triplet; q, quartet; sept, septet; m, multiplet), coupling constant(s) *J* (Hz), number of protons]. Analytical HPLC was performed using an UltiMate HPLC system consisting of an LPG‐3400A pump (1 mL/min), a WPS‐3000SL autosampler, and a 3000 Diode Array Detector installed with a Gemini‐NX C18 (250 × 4.60 mm, 3 µm) column. Solvent A: H_2_O + 0.1% TFA; Solvent B: MeCN‐H_2_O 9:1 + 0.1% TFA. For HPLC control, data collection, and data handling, the Chromeleon software v. 6.80 was used. Mass spectrometric data were recorded using either a Liquid Chromatography‐mass spectrometry (LC‐MS) system built from an Agilent 1200 series solvent delivery system equipped with an autoinjector coupled to a DAD and an Agilent 6130 A series quadrupole electrospray ionization detector. Solvent A: 5% aq MeCN + 0.1% HCO_2_H; Solvent B: MeCN + 0.1% HCO_2_H. Usually, gradients from A:B 1:0 to 0:1 (5 min). For data collection and data handling, the MassLynx software was used. The purity of compounds submitted for pharmacological characterization was determined by HPLC to be > 95%. CPX was obtained from Fluorochem Ltd (Hadfield, UK).

#### 2.1.1 1‐cyclopropyl‐6‐fluoro‐4‐oxo‐7‐(4‐((4‐(trifluoromethyl)phenyl)carbamothioyl)piperazin‐1‐yl)‐1,4‐dihydroquinoline‐3‐carboxylic acid (IMP‐1700)

This compound was synthesized as previously described with some minor modifications (Farooqi et al. 2019). CPX (250 mg, 0.75 mmol, 1.0 equiv.) was dissolved in dry DMF (40 mL), followed by the addition of NaHCO_3_ (76 mg, 0.90 mmol, 1.2 equiv.) and 4‐(trifluoromethyl) phenyl isothiocyanate (153 mg, 0.75 mmol, 1.0 equiv.) and the reaction was stirred for 17 h at room temperature. The reaction was quenched with sat. NH_4_Cl_(aq.)_ solution (25 mL) and was extracted using EtOAc (3 × 50 mL). The organic phase was washed with H_2_O (3 × 150 mL) and with brine (100 mL) and dried over MgSO_4_. After concentrating the organic phase, the crude was washed with H_2_O (2 × 50 mL) and MeOH (2 × 50 mL), which yielded the desired product as an off‐white solid (289 mg, 0.54 mmol, 72%).

**Figure A1:**
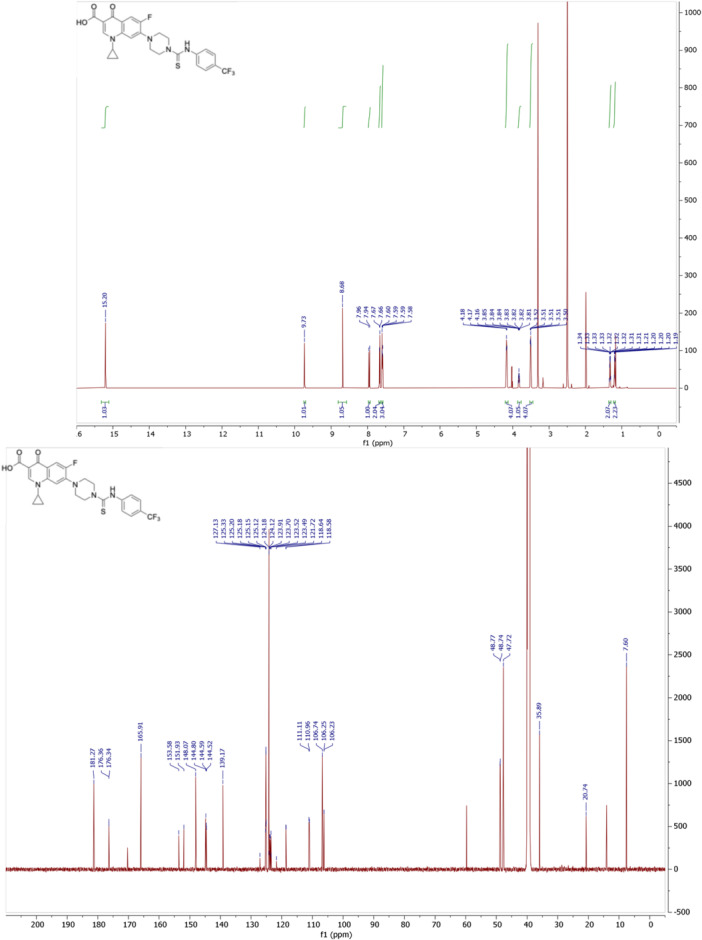
^1^H‐ and ^13^C‐NMR data of IMP‐1700.

**Figure A2:**
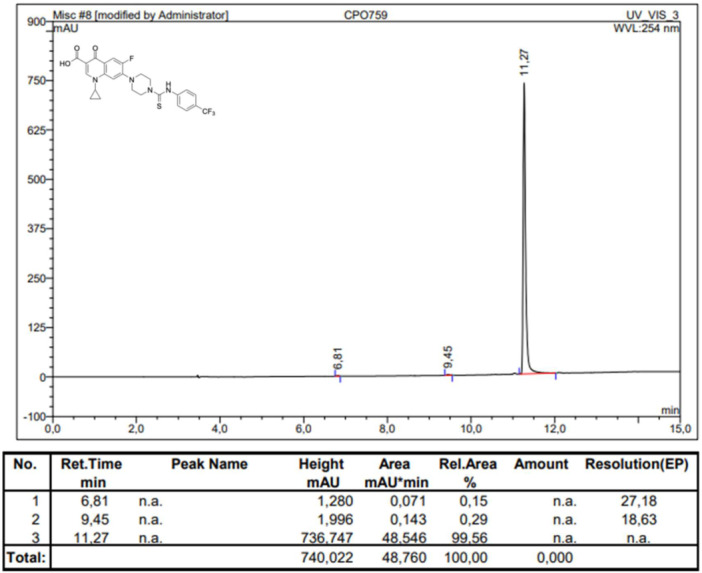
Analytical HPLC UV VIS data of IMP‐1700.

LCMS (ESI) m/z = 533.1 [M ‐ H]^‐^; ^1^H NMR (600 MHz, DMSO) δ 15.20 (s, 1H), 9.73 (s, 1H), 8.68 (s, 1H), 7.95 (d, *J* = 13.1 Hz, 1H), 7.66 (d, *J* = 8.3 Hz, 2H), 7.61 – 7.57 (m, 3H), 4.17 (t, *J* = 5.1 Hz, 4H), 3.83 (tt, *J* = 7.3, 4.0 Hz, 1H), 3.54 – 3.45 (m, 4H), 1.36 – 1.30 (m, 2H), 1.22 – 1.18 (m, 2H); ^13^C NMR (151 MHz, DMSO) δ 181.3, 176.4 (d, *J* = 2.4 Hz), 165.9, 152.8 (d, *J* = 249.2 Hz), 148.1, 144.8, 144.6 (d, *J* = 10.0 Hz), 139.2, 125.2 (d, *J* = 3.8 Hz), 124.4 (q, *J* = 271.4 Hz), 124,2, 123.8 (q, *J* = 31.9 Hz), 118.6 (d, *J* = 7.6 Hz), 111.0 (d, *J* = 23.1 Hz), 106.7, 106.2 (d, *J* = 3.4 Hz), 48.8 (d, *J* = 4.8 Hz), 47.7, 35.9, 20.7, 7.6. Data from NMR and HPLC are provided in Appendix Figures A1 and A2).

### 2.2 Bacterial Growth and Treatment

The bacterial strains of *S. aureus* (ATCC 29213, MH‐1670, and NCTC 83259) were cultivated in brain heart infusion (BHI) media at 37°C with shaking at 200 rpm. The bacterial suspension was centrifuged and adjusted to an optical density (OD) of 1.43, corresponding to approximately 1 × 10^9^ cells/mL, as measured using a spectrophotometer (Eppendorf BioPhotometer, Germany). Serial dilutions were prepared in phosphate‐buffered saline (PBS) in 1.5 mL Eppendorf tubes, ranging from 1 × 10^8^ to 1 × 10^3^ cells/mL for minimum inhibitory concentrations (MIC) assays. For drug treatment experiments, bacterial suspensions were treated with the indicated compounds before being subjected to serial dilution and plating.

MIC assays were performed by exposing bacteria to increasing concentrations of IMP‐1700, followed by incubation and enumeration of viable bacteria by colony counting on blood agar plates. MIC values were estimated by fitting the data to a Gompertz function (Lambert and Pearson 2000) using GraphPad Prism (version 10, GraphPad Software, San Diego, CA, USA):

y=A⋅exp[−exp(B(x−M))],

where *A* represents the lower asymptote, *B* the slope of the curve, and *M* the log‐transformed concentration at the inflection point.

The MIC was calculated as:

$$\mathrm{MIC}=10^{\left(M+1/B\right)}.$$

Minimum bactericidal concentration (MBC) was defined as the lowest concentration of IMP‐1700 that resulted in no visible bacterial growth on blood agar plates.

For combination and irradiation experiments, bacteria at a concentration of 1 × 10^5^ CFU/mL were exposed to varying concentrations of IMP‐1700 (0.025‐50 µM), CPX (10‐100 µM), and/or (2‐10 Gy) irradiation using an X‐RAD 320 (Precision X‐ray Irradiation, North Branford), operated at 320 kV and 12.5 mA. Irradiation time was regulated to deliver 1 Gy per minute (e.g., 120 s for 2 Gy). Treatments were administered either as monotherapy or in combination. IMP‐1700 was dissolved in ≥ 99.7% dimethyl sulfoxide (DMSO), and an equivalent volume of DMSO was included as a vehicle control.

Following treatment, a dilution series ranging from 10^5^–10^1^ was prepared in PBS, and bacterial suspensions from dilutions 10^4^–10^1^ were plated on blood agar plates (Herlev Hospital, Denmark). The plates were incubated at 37°C for 24 h, after which colony‐forming units (CFUs) were counted to assess bacterial survival.

### 2.3 Cell Cultivation and Treatment

The tumor cell lines, mouse melanoma B16.F10 (CRL‐6475, ATCC), human breast cancer MB‐MDA‐231 (CRM‐HTB‐26, ATCC), human hepatocellular carcinoma HepG2 C3A (a derivative of HepG2, CRL‐3581, ATCC), as well as the noncancerous mouse fibroblast cell line NIH‐3T3 (CRL‐1658, ATCC), were cultivated at 37°C, 5% CO2. The B16.F10 cell line was cultivated in Roswell Park Memorial Institute (RPMI) (Gibco, Thermo Fisher) supplemented with 10% Fetal Bovine Serum (FBS) (Gibco) and 1% Penicillin‐Streptomycin (pen/strep) (10,000 U/mL, Gibco), MB‐MDA‐231 was cultivated in MEM+ supplemented with 1% l‐Glutamine 100 × 200 mM (VWR), 1% SP, 1% MEM Non‐Essential Amino Acids Solution (100X) (Gibco), 10% FBS, and 1% pen/strep, and HepG2 cells were cultivated in EMEM + L‐Glutamine with 10% FBS and 1% pen/strep. NIH‐3T3 cells were cultivated in DMEM + L‐Glutamine supplemented with 10% FBS and 1% pen/strep.

The cells were harvested using TrypLE Express Enzyme (1X)(Gibco), and 100,000 cells/well were seeded in a 6‐well plate on the day of the experiments.

For each cell line, different treatments were conducted, including treatment with 5 µM IMP‐1700, 15 µM CPX, and irradiation with 10 Gy, as well as the combination of them all. The solvent for IMP‐1700 was DMSO, and as a control, DMSO was included with the same volume as IMP‐1700. The cell lines were incubated at three different time points after treatment (24, 48, and 72 h). To determine any changes in cell confluency and morphology, images were acquired for each treatment using the EVOS M5000 Imaging System with a 10X objective (Thermo Fisher). To quantify the cells and their viability after treatment, they were harvested using TrypLE and counted using the Countess II Automated Cell Counter (Thermo Fisher) using Erythrosin B Stain (Thermo Fisher). The total number of cells, the viability in percent, and the number of live cells were noted and used for data analysis.

### 2.4 Data Analysis and Statistical Analysis

Colony‐forming units per mL (CFU/mL) were calculated using the formula:

CFU/mL=(average count⋅dilution factor)/(plated volume in mL).

Experiments with *S. aureus* were performed with *n* = 4–8 biological replicates (exact n indicated in the figures), while mammalian cell experiments were conducted in triplicate (*n* = 3). Data are presented as mean ± standard deviation (SD). The fold changes were calculated relative to the untreated *S. aureus* control.

Dose‐response data were analyzed using GraphPad Prism (version 10; GraphPad Software, USA). LD_50_ values were obtained by fitting the data to a four‐parameter logistic (4PL) model with variable slope, defined as:

$$Y=\mathrm{Bottom}+\left(\mathrm{Top}-\mathrm{Bottom}\right)/(1+10^{\left(\mathrm{logLD}50-X\right)\cdot \mathrm{HillSlope})}),$$

where Y is the measured response, X is log_10_(dose), Top and Bottom represent the upper and lower plateau, and Hillslope describes the steepness of the curve. Percent inhibition was calculated as:

%Inhibition=(Top−Y)/(Top−Bottom)⋅100

Because near‐maximal inhibition was achieved at the highest tested dose, LD₉₀ was estimated by linear interpolation of experimental data points, bracketing 90% inhibition rather than by model extrapolation.

For combinations of IMP‐1700 and ciprofloxacin or irradiation, synergy was assessed using Bliss independence. Fractional inhibition values (0–1) were calculated for each single‐agent treatment (E_A_ = inhibition by drug A, E_B_ = inhibition by drug B), and the predicted inhibition for combined treatment was computed as:

$$E_{\mathrm{Bliss}}=E_{A}+E_{B}-\left(E_{A}\cdot E_{B}\right)$$

Observed inhibition of the combination was then compared to the Bliss‐predicted value to classify interactions as positive (synergistic) or negative (antagonistic). This analysis is presented in Appendix Table A3.

Statistical analyses were performed using GraphPad Prism (version 10). Differences between treatments were assessed using two‐way ANOVA, followed by Tukey's post hoc test, with α = 0.05. Statistical significance was defined as *p* < 0.05 (*), *p* < 0.005 (**), *p* < 0.0005 (***), and *p* < 0.0001 (****).

## 3 Results

### 3.1 Antibacterial Activity of Ciprofloxacin and IMP‐1700 in *S. aureus*

**Figure 2:**
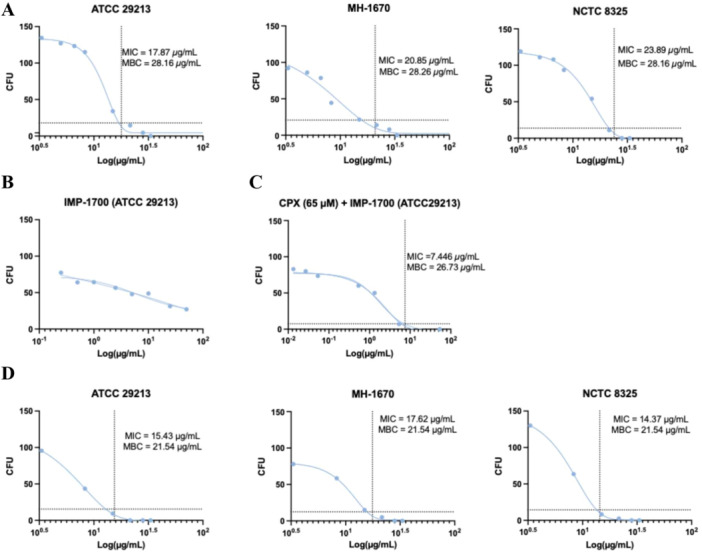
Antibacterial assays of CPX and IMP‐1700 against *S. aureus*. (A) CPX tested at 10–100 µM against strains ATCC 29213, NCTC 8325, and MH‐1670; MIC and MBC values determined. (B) IMP‐1700 tested as a single agent in ATCC 29213 over 0.0025–50 µM. (C) IMP‐1700 tested in combination with CPX (65 µM) in ATCC 29213; MIC and MBC determined. (D) CPX tested at 10–100 µM in combination with IMP‐1700 (5 µM) across all three strains. LogMIC was fitted to the Gompertz function using GraphPad Prism. Experiments were performed in *n* = 4. Full datasets can be found in Appendix Tables A1, A2.

Minimum inhibitory concentration (MIC) assays were conducted to assess the susceptibility of *S. aureus strains* ATCC 29213, MH‐1670, and NCTC 8325 to CPX (Figure 2A). CPX exposure (10–100 µM) yielded MIC values of 17.87 µg/mL, 20.85 µg/mL, and 23.89 µg/mL, for ATCC 29213, MH‐1670, and NCTC 8325, respectively. The minimum bactericidal concentration (MBC) was identical across all three strains (28.16 µg/mL). Dose‐response analysis enabled calculation of LD_50_ and LD_90_ values for CPX monotherapy: ATCC 29213, LD_50_ = 12.01 µM, LD_90_ = 19.4 µM; MH‐1670, LD_50_ = 8.68 µM, LD_90_ = 19 µM; NCTC 8325, LD_50_ = 13.97 µM, LD_90_ = 25.3 µM (Table 1). Given the comparable MIC, MBC, and LD values observed across strains, ATCC 29213 was selected as a representative strain for subsequent experiments.

**Table 1:** LD₅₀ and LD₉₀ values of ciprofloxacin (CPX) alone and in combination with IMP‐1700 against *S. aureus* strains.

Treatment	Strain	LD_50_ (µM)	LD_90_ (µM)
CPX	ATCC 29213	12.01	19.4
CPX	MH‐1670	8.68	19
CPX	NCTC 8325	13.97	25.3
CPX + IMP‐1700 (5 µM)	ATCC 29213	7.7	14.8
CPX + IMP‐1700 (5 µM)	MH‐1670	4.11	8.2
CPX + IMP‐1700 (5 µM)	NCTC 8325	8.13	13.4

IMP‐1700 was next evaluated as a single agent against *S. aureus* ATCC 29213 over a concentration range of 0.0025–50 µM (Figure 2B). Although a concentration‐dependent reduction in bacterial growth was observed, no MIC could be determined.

To assess potential combinatorial effects, IMP‐1700 (0.025–100 µM) was tested in the presence of a fixed concentration of CPX (65 µM) (Figure 2C). Under these conditions, an MIC of 7.446 µg/mL and an MBC of 26.73 µg/mL were determined.

Based on these findings, a sub‐inhibitory concentration of IMP‐1700 (5 µM) was selected for combination studies across all three *S. aureus* strains. CPX (10–100 µM) was conducted in combination with IMP‐1700 across all three *S. aureus* strains (Figure 2D, Table 1). The resulting MIC and MBC values were as follows: ATCC 29213 (MIC of 15.43 µg/mL, MBC of 21.54 µg/mL), MH‐1670 (MIC 17.62 µg/mL, MBC 21.54 µg/mL), and NCTC 8325 (MIC 14.37 µg/mL, MBC of 21.54 µg/mL). Dose‐response analysis for the combination allowed calculation of LD_50_ and LD_90_ values: ATCC 29213, LD_50_ = 7.7 µM, LD_90_ = 14.8 µM; MH‐1670, LD_50_ = 4.11 µM, LD_90_ = 8.2 µM; NCTC 8325, LD_50_ = 8.13 µM, LD_90_ = 13.4 µM (Table 1). Collectively, these results indicate a strain‐independent synergistic interaction between IMP‐1700 and CPX.

### 3.2 IMP‐1700 and Ciprofloxacin Exhibit Synergistic Antibacterial Activity

**Figure 3:**
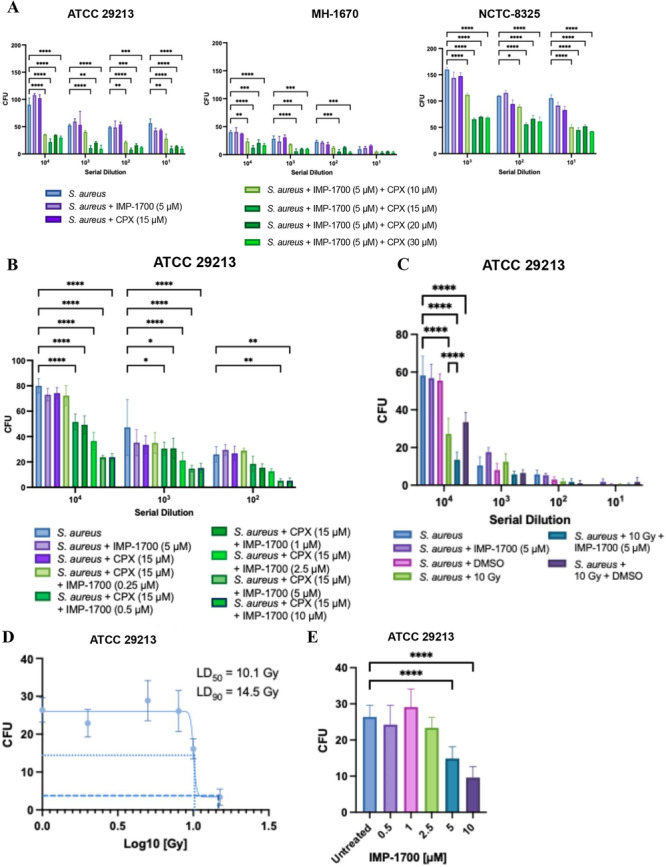
(A) Growth of three S. aureus strains (ATCC 29213, MH‐1670, NCTC 8325) after treatment with IMP‐1700, ciprofloxacin (CPX), and their combination, quantified by colony‐forming units (CFU) across serial dilutions. (B) Dose‐response of IMP‐1700 in combination with a fixed CPX concentration in ATCC 29213, measured via serial dilution (10^4^–10^2^ CFU). (C) Serial dilution of S. aureus ATCC 29213 following combination treatments with and without X‐ray exposure (10 Gy). Shown as a dilution series from 10^4^ to 10^1^ CFU. IMP‐1700 was used at 5 µM diluted in DMSO, and DMSO was added at an equivalent volume. (D) Viability of S. aureus ATCC 29213 following X‐ray exposure (2–15 Gy), quantified by CFU at 10^4^ dilution. LD_50_ (dashed line) was determined from the Prism‐fitted curve, and LD_90_ (long dashed line) was estimated by linear interpolation of the experimental data. (E) Viability of S. aureus ATCC 29213 following X‐ray exposure in combination with IMP‐1700 (0.5–10 µM), quantified by CFU at 10^4^dilution. All counts were performed on blood agar plates. Error bars represent standard deviations, with significance determined by a two‐way ANOVA with Tukey's post hoc test. Values only shown compared to S. aureus untreated or 10 Gy: *p* < 0.05 (*), *p* < 0.005 (**), *p* < 0.0005 (***), *p* < 0.0001 (****), *n =* 8. Full datasets can be found Appendix Tables A3, A4.

To further characterize the antibacterial synergy, *S. aureus* strains were treated with IMP‐1700 and CPX as monotherapy or in combination to assess the antibacterial effects. As shown in Figure 3A,B, neither IMP‐1700 nor CPX alone produced a significant reduction in bacterial viability compared with untreated controls. In contrast, combination treatment significantly reduced bacterial viability (*p* = 0.0242 to < 0.0001), and Bliss independence analysis confirmed a synergistic interaction across all tested strains (Appendix Tables A1 and A3). These results confirm previously reported synergistic antibacterial activity of IMP‐1700 and CPX combinations in *S. aureus* (Lim et al. 2019), while providing new insights into the dose response of IMP‐1700. Specifically, 5 µM IMP‐1700 is sufficient for maximal efficacy in combination therapy of 15 µM CPX, consistent with MIC assay results. Inclusion of multiple *S. aureus* strains and for serial dilution assays adds translational relevance to earlier single‐strain studies (Lim et al. 2019). The ATCC 29213 strain was prioritized for further work due to its prevalence in the current literature.

As shown in Figure 3C, IMP‐1700 (5 µM) further sensitized *S. aureus* to X‐ray‐induced bacterial killing. Combined treatment with IMP‐1700 and 10 Gy X‐ray irradiation resulted in a pronounced reduction in bacterial viability (*p* < 0.0001). DMSO controls exhibited no effect in either irradiated or non‐irradiated conditions, confirming treatment specificity. Quantitative CFU/mL analysis revealed a 77% decrease in bacterial survival with combination treatment (13.5 × 10^6^ CFU/mL ± 4.12 × 10^6^) compared with untreated controls (58.25 × 10^6^ CFU/mL ± 10.18 × 10^6^), and Bliss independence analysis indicated a positive interaction between IMP‐1700 and irradiation (Appendix Tables A2, A3).

Exposure to X‐ray monotherapy (2–15 Gy) produced a dose‐dependent reduction in *S. aureus* ATCC 29213 viability (Figure 3D), with an LD_50_ of 10.0 Gy and an LD_90_ of approximately 14.5 Gy. When increasing concentrations of IMP‐1700 (0.5–10 µM) in combination with X‐ray exposure it further reduced bacterial viability, with significant effects demonstrated at 5 and 10 µM (*p* < 0.0001) (Figure 3E). These results demonstrate that IMP‐1700 not only potentiates CPX‐mediated antibacterial activity but also enhances bacterial sensitivity to irradiation‐induced stress, highlighting its potential as a combinatorial adjuvant.

### 3.3 X‐Ray Reduces Viability of Mammalian Cells With Cell‐Type Specific Sensitivity

**Figure 4:**
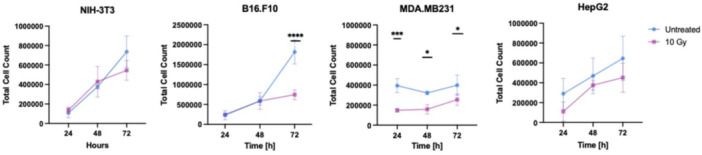
Effect of 10 Gy X‐ray on viability of NIH‐3T3, B16.F10, MDA‐MB‐231, and HepG2 cells. Blue: untreated controls; Pink: irradiated cells. Cell counts were conducted at 24, 48, and 72 h post‐treatment. Statistical analysis was performed using two‐way ANOVA with Tukey's post hoc test: *p* < 0.05 (*), *p* < 0.005 (**), *p* < 0.0005 (***), *p* < 0.0001 (****), *n* = 3. Note: *Y*‐axes are scaled independently for each cell line to account for differences in baseline growth, allowing clearer visualization of treatment effects. Tumor cell counts and representative images can be found in Appendix Table A4 and Figure A3.

**Figure A3:**
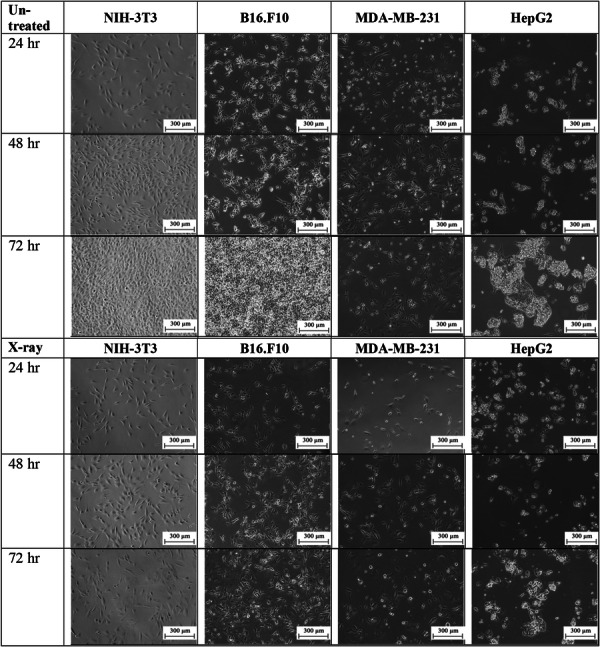
Images of untreated NIH‐3T3, B16.F10, MDA‐MB‐231, and HepG2 cells and cells exposed to X‐ray. Scalebar: 300 µm.

The impact of 10 Gy X‐ray exposure was evaluated in the noncancerous fibroblast cell line NIH‐3T3 and tumor cell lines B16.F10, MDA‐MB‐231, and HepG2. As shown in Figure 4, the NIH‐3T3 fibroblasts were minimally affected, showing fold changes of 1.26, 1.14, and 0.74 at 24, 48, and 72 h, respectively (Appendix Table A4 and Figure A3).

Among tumor cell lines, the B16.F10 cell counts were significantly reduced only at 72 h (*p* < 0.0001), whereas MDA‐MB‐231 cells showed significant reduction at all time points (24 h: *p* = 0.0008; 48 h: *p* = 0.0164; 72 h: *p* = 0.0330). In contrast, HepG2 cells demonstrated a limited response to radiation.

B16.F10 cells showed a delayed response to irradiation, with a pronounced reduction in viability observed at 72 h (0.41‐fold). In contrast, MDA‐MB‐231 cells exhibited consistent radiosensitivity across all time points, with fold changes of 0.46, 0.40, and 0.64 at 24, 48, and 72 h, respectively. HepG2 cells were comparatively more radioresistant, displaying a modest reduction at 24 h (fold change 0.38), followed by partial recovery at later time points (Appendix Table A4). Overall, while some degree of post‐irradiation recovery was evident in all cell lines, these responses were highly cell‐type dependent, underscoring substantial heterogeneity in tumor radiosensitivity.

### 3.4 IMP‐1700 Enhances Radiation‐Induced Mammalian Cell Death

**Figure 5:**
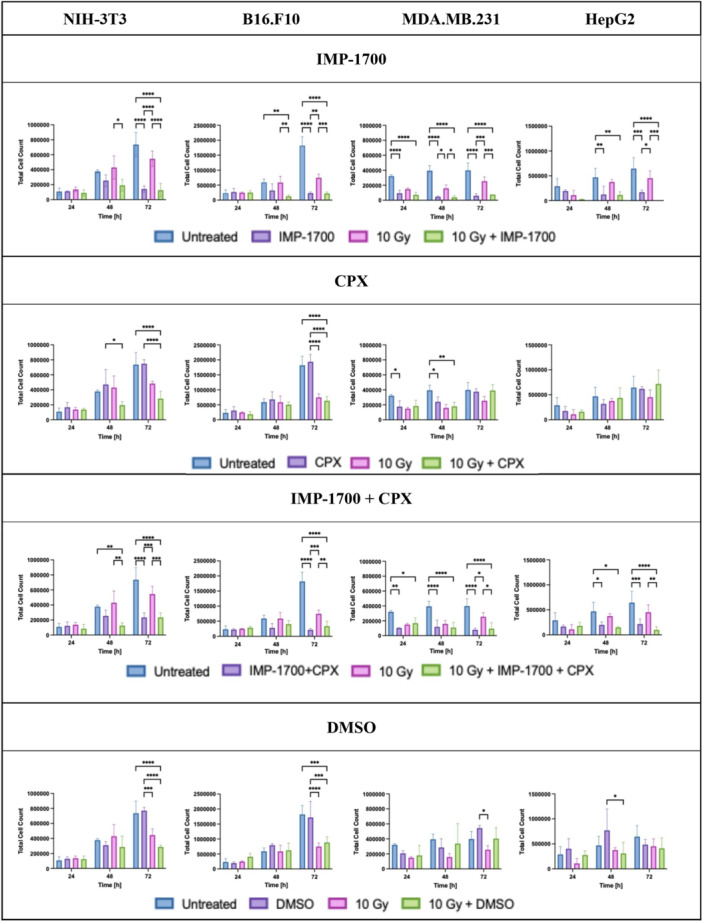
Viability of NIH‐3T3, B16.F10, MDA‐MB‐231, and HepG2 cells after treatment with IMP‐1700 (5 µM), ciprofloxacin (CPX) (15 µM), their combination, or DMSO, with and without 10 Gy X‐ray. Cell counts were measured at 24, 48, and 72 h post‐treatment. Statistical significance assessed via two‐way ANOVA and Tukey's post hoc test: *p* < 0.05 (*), *p* < 0.005 (**), *p* < 0.0005 (***), *p* < 0.0001 (****), *n* = 3. Significant differences are only shown compared to untreated or 10 Gy. Tumor cell count values and images are available in Appendix Table A5 and Figure A4, A5, A6, A7.

**Figure A4:**
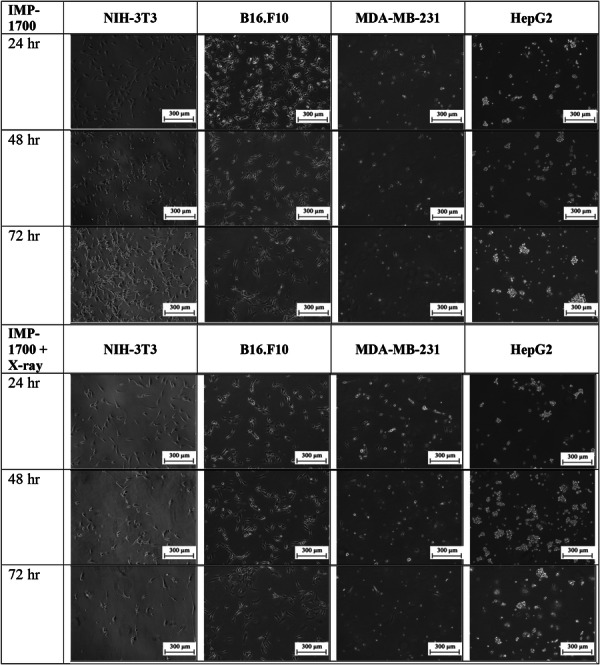
Images of NIH‐3T3, B16.F10, MDA‐MB‐231, and HepG2 cells treated with IMP‐1700, with and without X‐ray. Scalebar: 300 µm.

To determine whether IMP‐1700 enhances radiosensitivity in cells, a study measuring the viability was performed following treatment with IMP‐1700 as monotherapy or in combination with X‐ray irradiation (Figure 5, Appendix Tables A4 and A5 and Figure A4). For NIH‐3T3 cells, the combined therapy significantly reduced viability at 48 h compared with X‐ray monotherapy (*p* = 0.0120) and at 72 h compared with both untreated controls and X‐ray monotherapy (*p* < 0.0001). The fold changes for IMP‐1700 monotherapy were 1.03, 0.68, and 0.19, decreasing to 0.84, 0.51, and 0.17 for the combination therapy at 24, 48, and 72 h, respectively. In B16.F10 cells, combination therapy significantly reduced viability compared with single treatments at both 48 h (*p* = 0.0032 to 0.0035) and 72 h (*p* = 0.0009 to < 0.0001). The fold changes in viability for B16.F10 with IMP‐1700 monotherapy were 1.16, 0.54, and 0.13 at 24, 48, and 72 h, respectively, while the addition of 10 Gy reduced this further to 1.09, 0.21, and 0.12. MDA‐MB‐231 cells showed similar radiosensitisation, with IMP‐1700 as monotherapy resulting in fold changes of 0.29, 0.12, and 0.15, and combination treatment achieving 0.22, 0.10, and 0.19 at the same time points. HepG2 cells, although initially resistant, demonstrated significant viability loss at 48 and 72 h (*p* = 0.0394 to < 0.0003), with fold changes for IMP‐1700 monotherapy of 0.67, 0.26, and 0.26, and combination therapy with 10 Gy dropping to 0.09, 0.24, and 0.00. No treatment group showed increased cell counts over time, indicating decreased cell division and sustained cytotoxic effects. These results support the radiosensitizing effect of IMP‐1700 in various tumor cell lines.

**Figure A5:**
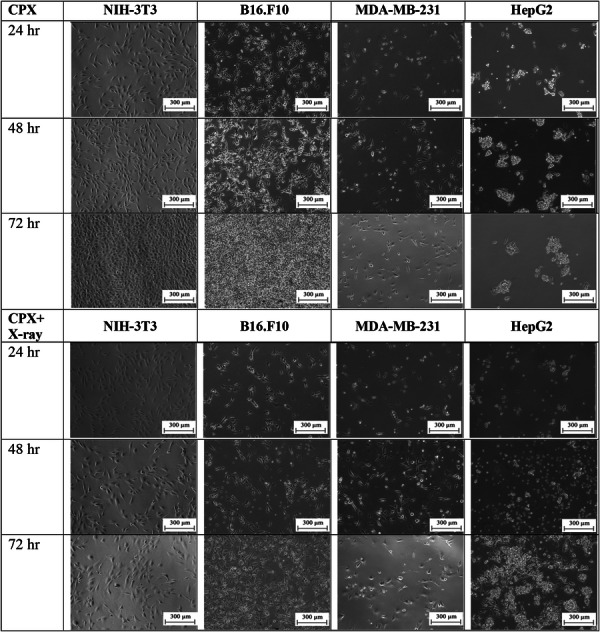
Images of NIH‐3T3, B16.F10, MDA‐MB‐231, and HepG2 cells treated with ciprofloxacin (CPX), with and without X‐ray. Scalebar: 300 µm.

CPX treatment caused a limited reduction in cell viability (Figure 5, Appendix Tables A4 and A5 and Figure A5). For NIH‐3T3 cells, CPX monotherapy resulted in fold changes of 1.55, 1.25, and 1.06, decreasing to 1.26, 0.52, and 0.38 with combination treatment at 24, 48, and 72 h, respectively. In B16.F10 cells, fold changes for CPX as monotherapy were 1.33, 1.15, and 1.43 at 24, 48, and 72 h, respectively, which decreased to 0.81, 0.86, and 0.35 when combined with radiation. MDA‐MB‐231 cells exhibited fold changes of 0.55, 0.61, and 0.94 with CPX as monotherapy, and 0.57, 0.45, and 0.98 with added radiation. A similar trend was observed in HepG2 cells, with CPX monotherapy resulting in values of 0.61, 0.68, and 0.96, and 0.55, 0.93, and 1.11 when combined with X‐ray. Both MDA‐MB‐231 and HepG2 cells demonstrated increased cell growth over time, while the viability of the B16.F10 cells decreased at 72 h.

**Figure A6:**
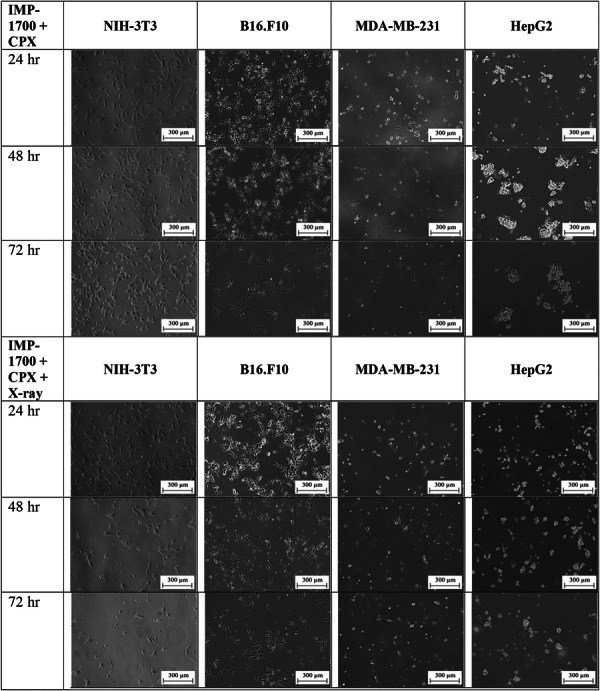
Images of NIH‐3T3, B16.F10, MDA‐MB‐231, and HepG2 cells treated with IMP‐1700 and ciprofloxacin (CPX), with and without X‐ray. Scalebar: 300 µm.

To evaluate any potential synergistic cytotoxicity, the four cell lines were treated with IMP‐1700 and CPX, with or without X‐ray irradiation (Figure 5, Appendix Tables A4 and A5 and Figure A6). In NIH‐3T3 cells, the combination treatment with X‐ray significantly reduced viability at both 48 and 72 h. At 48 h, viability was in untreated and X‐ray‐only controls (*p* = 0.0066 and *p* = 0.0010, respectively), while at 72 h, similar reductions were seen compared with untreated cells and X‐ray monotherapy (*p* < 0.0001 and *p* = 0.0008). Fold changes for the combination treatment were 1.13, 0.68, and 0.32 without radiation, further decreasing to 0.77, 0.33, and 0.32 with X‐ray at 24, 48, and 72 h, respectively. B16.F10 cells exhibited significant reductions in viability at 72 h (*p* = 0.0085 to < 0.0001), with fold changes of 0.96, 0.48, and 0.12 for the combination without radiation and 1.23, 0.68, and 0.18 with radiation, at 24, 48, and 72 h, respectively. MDA‐MB‐231 cells showed consistent sensitivity across all time points for the combinatorial treatment without radiation, with fold changes of 0.32, 0.30, and 0.20 without radiation, and 0.52, 0.28, and 0.24 with X‐ray at 24, 48, and 72 h, respectively. For the HepG2 cells, viability following combination treatment decreased to 0.57, 0.42, and 0.34 without radiation, and further to 0.62, 0.32, and 0.15 with X‐ray at 24, 48, and 72 h, respectively. These findings demonstrate a cell‐type‐dependent reduction in viability across all three cell lines following combination treatment, with enhanced effects detected over time and with the addition of radiation.

**Figure A7:**
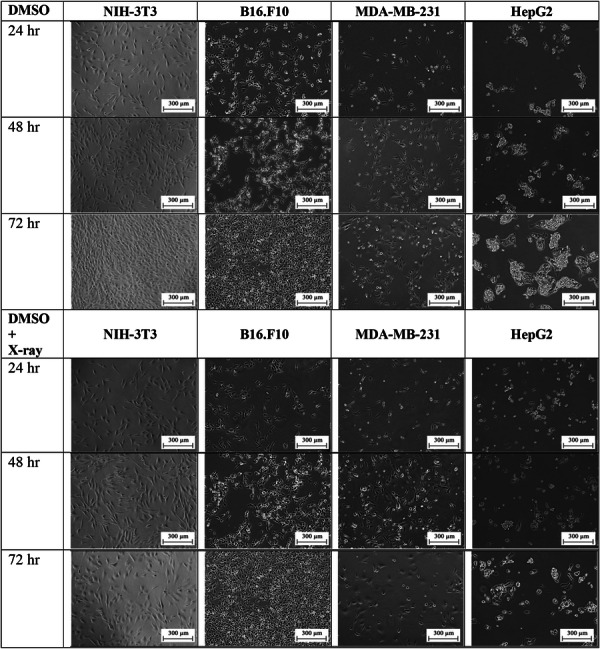
Images of NIH‐3T3, B16.F10, MDA‐MB‐231, and HepG2 cells treated with DMSO, with and without X‐ray. Scalebar: 300 µm.

DMSO was included as a vehicle control, since IMP‐1700 was dissolved in DMSO, and tested in parallel to account for any solvent‐related effects (Figure 5, Appendix Tables A4 and A5 and Figure A7). Treatment with DMSO as monotherapy did not significantly impact cell viability, aside from expected differences between irradiated and non‐irradiated groups, confirming the lack of cytotoxicity from the solvent.

## 4 Discussion

The rise of antimicrobial resistance demands innovative strategies to enhance existing therapies and develop novel therapeutics. Traditional antibiotics like CPX are increasingly compromised by resistance mechanisms. One promising strategy involves interfering with bacterial responses to genotoxic stress, which are critical for survival following DNA damage. In this study, IMP‐1700 was investigated as a compound designed to modulate bacterial DNA damage‐associated pathways and thereby sensitize *S. aureus* to CPX and radiation‐induced stress, while assessing potential off‐target effects in mammalian cells.

The combination of IMP‐1700 and CPX demonstrated a strong synergistic antibacterial effect in *S. aureus*, confirming and extending previous findings (Lim et al. 2019). While CPX monotherapy exhibited consistent MIC and MBC values across *S. aureus* strains, IMP‐1700 showed no intrinsic antibacterial activity, supporting its role as an adjuvant rather than a standalone antimicrobial. Co‐treatment with IMP‐1700 significantly reduced CPX MIC values and shifted the LD_50_/LD_90_ values toward lower concentrations across all strains. The lack of a strictly dose‐dependent response to increasing CPX concentrations when IMP‐1700 was maintained at a fixed sub‐inhibitory level likely reflects early saturation of antibacterial killing once bacterial tolerance to genotoxic stress is reduced. This interpretation is consistent with previous work showing that CPX's bactericidal effect can be enhanced when responses to DNA damage are disrupted (Lim et al. 2019).

Consistent with the MIC and MBC findings, dose‐response analysis showed that IMP‐1700 markedly enhanced CPX efficacy across all tested *S. aureus* strains. Combination treatment produced a leftward shift of the dose‐response curves, indicating that lower CPX concentrations were required to achieve equivalent levels of bacterial killing. Formal Bliss independence analysis confirmed that the interaction between IMP‐1700 and CPX was synergistic across all tested *S. aureus* strains (Appendix Table A3), supporting the conclusion that IMP‐1700 acts as a sensitizer rather than a standalone antimicrobial. This effect was observed consistently across genetically distinct strains, supporting a non‐strain‐specific sensitizing effect of IMP‐1700 rather than a strain‐specific interaction.

X‐ray monotherapy produced a dose‐dependent reduction in bacterial viability, consistent with previous reports describing the relative radioresistance of *S. aureus* (Adebiyi et al. 2020). Dose‐response analysis revealed an LD_50_ of approximately 10 Gy and an LD_90_ of ~14.5 Gy, underscoring the requirement for relatively high radiation doses to achieve substantial bacterial killing. Importantly, treatment with IMP‐1700 shifted the dose‐response relationship toward enhanced radiosensitivity, resulting in further reductions in bacterial survival at concentrations of 5‐10 µM. Notably, 5 µM IMP‐1700 not only acted synergistically with 15 µM CPX but also increased bacterial sensitivity to irradiation‐induced stress. The consistency of these effects across multiple *S. aureus* strains supports a strain‐independent sensitizing effect of IMP‐1700. Bliss independence analysis similarly indicated a positive interaction between IMP‐1700 and X‐ray irradiation (Appendix Table A3), consistent with the observed enhancement of bacterial radiosensitivity.

Furthermore, combining IMP‐1700 with 10 Gy X‐ray irradiation resulted in a 77% reduction in bacterial survival. While the underlying mechanisms were not directly assessed, these findings suggest that IMP‐1700 can exacerbate bacterial vulnerability to genotoxic stress. Together, these results support the use of irradiation as a proof‐of‐concept tool to evaluate bacterial sensitization and provide a framework for exploring stress‐response–targeting adjuvants in combination with antimicrobial strategies.

To place these findings in context and clarify the rationale for the radiation experiments, X‐ray irradiation was used as a controlled method to induce DNA damage and assess whether IMP‐1700 could enhance bacterial killing. While X‐ray is not a standard antibacterial therapy, it provides a proof‐of‐concept tool and a bridge to mammalian cell experiments. Although IMP‐1700 was designed to target bacterial AddAB/RecBCD complexes that are absent in mammalian cells, the observed cytotoxicity in eukaryotic cells highlights that the compound is not fully selective at the concentrations tested. The specific DNA repair pathways affected by IMP‐1700 in combination with X‐ray were not directly measured, and no mechanistic conclusions were drawn. We note that the timing and dose of X‐ray irradiation relative to drug addition may influence bacterial responses; in this study, IMP‐1700 was added immediately after irradiation, and additional work will explore dose‐response and timing effects systematically.

To evaluate the selectivity of IMP‐1700, a diverse panel of mammalian cell lines was used: noncancerous fibroblast (NIH‐3T3), murine melanoma (B16.F10), triple‐negative breast cancer (MDA‐MB‐231), and human hepatocellular carcinoma (HepG2).

When exposed to X‐ray alone, NIH‐3T3 cells exhibited no significant change in viability compared with untreated controls, consistent with reports that non‐transformed cells exhibit relative resistance to X‐ray–induced damage due to intact DNA repair and cell‐cycle checkpoint mechanisms (Miller et al. 1985; Pirollo et al. 1993; Rodier et al. 2009). Fibroblasts, in particular, preferentially undergo cell‐cycle arrest and DNA repair rather than apoptosis following irradiation (Pucci et al. 2000). In line with this, Yamamori et al. (2017) demonstrated that irradiated NIH‐3T3 cells remain metabolically active, with increased mitochondrial mass and mtDNA content, while entering long‐term cell‐cycle arrest. (Yamamori et al. 2017). The tumor cell lines exhibited variable radiosensitivity (Figure 4). Among the cancer cell lines, B16.F10 cells showed significant reductions in cell viability at 72 h post‐irradiation, consistent with previous findings in human melanoma cells (ATCC CRL‐1619IG‐2) (Q. Zhang et al. 2020). Notably, MDA‐MB‐231 cells also demonstrated enhanced sensitivity to X‐ray radiation, consistent with recent findings by Mahmoud et al. (2023), who reported increased DNA damage markers and apoptosis in MDA‐MB‐231 cells when exposed to X‐rays. Their study reported significant upregulation of cleaved caspase‐3/7 and γH2AX foci at 24 h after 8.5 Gy irradiation, indicating that MDA‐MB‐231 cells possess moderate radiosensitivity that can be further potentiated under specific genotoxic stress conditions (Mahmoud et al. 2023). Additionally, Tochaikul et al. (2024) showed that even low doses of X‐ray irradiation could inhibit cell growth and viability in MDA‐MB‐231 cells, reinforcing the inherent radiosensitivity of this cell line (Tochaikul et al. 2024). In contrast, HepG2 cells displayed minimal response to X‐ray treatment, consistent with known radioresistance in hepatocellular carcinoma (Herold et al. 2002; Kuwahara et al. 2009). While Das et al. (2017) reported ROS‐mediated death in HepG2 cells post‐radiation, we did not observe this effect under our conditions. This discrepancy may reflect a greater radiation sensitivity of the HepG2 subline (ATCC, CRL‐119967) used in their study or different experimental parameters (Das et al. 2017). These observations confirm that radiosensitivity varies significantly between tumor types and highlight the need for tailored approaches when combining genotoxic stress‐sensitizing agents with radiotherapy.

In addition to its potent antibacterial effects, IMP‐1700 also exerted pronounced cytotoxic effects in both tumor and non‐tumor cell lines. Accordingly, all tested cell lines exhibited significant viability reduction following IMP‐1700 monotherapy, which further enhanced when combined with radiation (Figure 5).

While IMP‐1700 was originally developed as a selective inhibitor of bacterial DNA repair, targeting the AddAB/RecBCD pathways, our findings indicate that it may also influence cancer cells under specific conditions of genotoxic stress. Lim et al. (2019) demonstrated that IMP‐1700 sensitized *S. aureus* with CPX by inhibiting bacterial double‐strand break repair, with minimal toxicity seen in mammalian cells at bactericidal concentrations (Lim et al. 2019). The observed selectivity was thought to result from the absence of bacterial‐specific repair complexes in mammalian cells. However, in our study, IMP‐1700 exhibited cytotoxic effects in all cancer cell lines tested, which were further enhanced by radiation.

This lack of selectivity suggests that IMP‐1700 may affect cellular stress‐response pathways in mammalian cells, particularly under genotoxic conditions. While the specific molecular targets of IMP‐1700 in mammalian cells remain unknown, off‐target or indirect effects cannot be excluded. Further studies are needed to identify the basis of this toxicity profile and to guide optimization for selective antibacterial activity.

In our study, IMP‐1700 monotherapy caused a substantial reduction in NIH‐3T3 cell viability, with cell numbers decreasing by 81% after 72 h relative to untreated controls. Combination treatment with IMP‐1700 and X‐ray irradiation further reduced the viability by 83%, indicating that the compound enhances radiosensitisation even in healthy fibroblasts, which showed relative resistance to X‐ray treatment alone. For B16.F10 cells, IMP‐1700 monotherapy decreased viability by 87% at 72 h, with combination treatment producing a slightly greater reduction to 88%. In contrast, MDA‐MB‐231 cells exhibited enhanced sensitivity to IMP‐1700 already at 24 h, with cell viability decreasing by 71%, progressing to an 85% reduction at 72 h; combination treatment further enhanced this effect, with reductions ranging from 78% to 81% over the same period. These results confirm that IMP‐1700 effectively enhances radiosensitisation, though the specific DNA repair pathways affected were not directly measured. Notably, even HepG2 cells, described as resistant to radiation (Herold et al. 2002; Kuwahara et al. 2009), exhibited a decrease in viability from 91% at 24 h to complete loss of viability at 72 h under combined treatment. Together, these data indicate that IMP‐1700, both as monotherapy and in combination with X‐ray irradiation, significantly reduces cell viability in both healthy and cancerous cells. Whether the effects arise from direct cytotoxicity, indirect interference with DNA repair, increased oxidative stress, or other off‐target mechanisms, remains to be elucidated, as the specific DNA repair pathways were not directly measured in this study.

In contrast, CPX monotherapy demonstrated minimal effect on mammalian cell viability. As shown in Figure 5, cell viability remained largely stable across most cell lines and time points following CPX monotherapy. After 72 h post‐treatment, a fold change was observed with 1.06 for NIH‐3T3, 1.43 for B16.F10, 0.94 for MDA‐MB‐231, and 0.96 for HepG2, indicating modest to minimal effects. This limited effect may be due to the dosage used, as Kloskowski et al. (2011) reported a reduction in HepG2 cells' viability at concentrations starting from 25 µg/mL, a concentration five times higher than what was used in this study (Kloskowski et al. 2011). Combining CPX with radiation led to more pronounced reductions in cell viability, particularly in NIH‐3T3 cells (0.38‐fold at 72 h), B16.F10 cells (0.35‐fold at 72 h), and to a lesser extent in MDA‐MB‐231 cells (0.98‐fold at 72 h). These findings were not significantly different from tumor cells treated with X‐ray alone, indicating that CPX itself had no effect on the viability of the tumor cells at this concentration.

This deviates from the findings of Beberok et al. (2018), who reported that CPX as monotherapy could induce apoptosis in MDA‐MB‐231 cells via activation of the p53/Bax/Bcl‐2 signaling pathway (Beberok et al. 2018). In their study, CPX concentrations ranging from 0.0001 to 1 µmol/mL led to a 50% reduction in cell viability after 72 h of treatment with 0.01 µmol/mL compared to the control. Although our CPX concentration was in the range of Beberok et al. (2018), we did not observe a comparable cytotoxic effect. This discrepancy likely reflects differences in experimental conditions between our studies, such as CPX formulation, treatment media, assay type (e.g., MTT vs. live cell count), different cell lines, or subtle differences in cell line behavior under our conditions. The variation in the outcomes highlights how experimental context may have influenced the drug response.

The combination of IMP‐1700 and CPX demonstrated cytotoxic effects across all cell lines. In NIH‐3T3, the combination treatment significantly reduced the cell viability compared to the untreated controls and irradiated monotherapy. In B16.F10 cells, the combination caused a delayed reduction in viability with a significant decrease at 72 h. MDA‐MB‐231 cells displayed consistent sensitivity to IMP‐1700 as monotherapy, with limited further reduction in viability when combined with CPX or radiation, suggesting that IMP‐1700 was the primary driver of cytotoxicity in this line. In HepG2 cells, the combination of IMP‐1700 and CPX led to decreased viability; it did not differ from IMP‐1700 monotherapy, indicating no additive or synergistic interaction between the two compounds. These findings highlight the cytotoxic effect of IMP‐1700 across all cell types tested. While CPX contributes minimally to overall cell death, the addition of radiation may serve as an effective adjunct, particularly in treatment‐resistant tumor cells.

These findings confirmed IMP‐1700s antibacterial potency but raised significant concerns about mammalian toxicity. While other studies may report better selectivity, likely due to differences in cell models or experimental design, our broad cell panel underscored the need for further investigation. Further studies should clarify the mechanism of IMP‐1700 in mammalian cells, including whether the effects might involve DNA damage response, oxidative stress, and potential off‐target effects. In addition, future work should evaluate the activity of IMP‐1700 against other pathogenic species and commensal bacteria to better define its antimicrobial spectrum and selectivity.

## 5 Conclusion

This study demonstrates that IMP‐1700 has dual biological effects, potent antibacterial activity, and measurable cytotoxicity in mammalian cells. IMP‐1700 significantly enhanced the antibacterial efficacy of CPX and sensitized *S. aureus* to ionizing radiation. The observed synergy between IMP‐1700 and CPX across multiple *S. aureus* strains is consistent with previous findings and supports further exploration of bacterial DNA damage response‐associated pathways as therapeutic targets to overcome antibiotic resistance. Furthermore, combining IMP‐1700 with X‐ray irradiation resulted in marked reductions in bacterial viability, suggesting its utility in adjunct antimicrobial‐radiation therapies for resistant or localized infections.

In mammalian cells, IMP‐1700 reduced viability in NIH‐3T3 fibroblasts and tumor cell lines (B16.F10, MDA‐MB‐231, HepG2), both as monotherapy and in combination with radiation. While a transient increase in cytotoxicity was observed in B16.F10 cells at 48 h, similar effects were not consistently seen in other cell lines or time points. These findings indicate variable and time‐dependent effects in mammalian cells and underscore the compound's limited selectivity for bacterial targets at the concentrations tested.

Altogether, the results support the antibacterial potential of IMP‐1700 in enhancing sensitivity to genotoxic stress, while highlighting the need for caution regarding mammalian cytotoxicity. Further mechanistic studies are necessary to elucidate the basis of these effects and to improve the therapeutic window. Optimization of compound selectivity and delivery, potentially through targeted formulations or dose modulation, will be critical before considering clinical translation of IMP‐1700 as an antimicrobial or radiosensitising agent.

## Author Contributions

**Ida Vang Andersen:** conceptualization (lead), performing the experiment (lead), analyzing the data (lead), writing – original draft (lead), writing – review and editing (equal). **Ane Beth Sloth:** conceptualization (lead), performing the experiment (participated), writing – original draft (contributed), writing – review and editing (equal). **Emilie Caroline Skuladottir Bøgestad:** performing the experiment (participated), writing – review and editing (equal). **Christian Bernard Matthijs Poulie:** performing the experiment on compounds (lead), writing – original draft (contributed), writing – review and editing (equal); **Anne Skovsbo Clausen:** conceptualization (participated), writing – review and editing (equal). **Umberto Maria Battisti:** conceptualization (participated), writing – review and editing (equal); **Matthias M. Herth:** conceptualization (participated), writing – review and editing (equal), funding acquisition, supervision, resources; **Andreas Kjaer:** conceptualization (participated), writing – review and editing (equal), funding acquisition, supervision, resources.

## Ethics Statement

The authors have nothing to report.

## Conflicts of Interest

The authors declare no conflicts of interest.

## Data Availability

Raw data can be found in the Appendix.

## 1

1. **LCMS and HPLC results of IMP‐1700**

2. **Bacterial growth**

**Table A1:** Bacterial growth of ATCC 29213, MH‐1670, and NCTC 8325.

	Control	CPX (15 µM)	IMP‐1700 (5 µM)	IMP‐1700 10 µM CFX	IMP‐1700 15 µM CFX	IMP‐1700 + 20 µM CFX	IMP‐1700 + 30 µM CFX
**ATCC 29213**							
CFU/mL (10^4^)	9.05·10^7^	1.08·10^8^	1.03·10^8^	3.55·10^7^	2.20·10^7^	3.45·10^7^	3.00·10^7^
SD	2.12	2.83	6.36	0.71	5.66	0.71	2.83
Fold	1.00	1.34	1.27	0.44	0.27	0.43	0.37
CFU/mL (10^3^)	5.25·10^7^	5.95·10^7^	6.80·10^7^	4.05·10^7^	1.10·10^7^	2.05·10^7^	9.50·10^6^
SD	2.12	4.95	4.24	2.12	4.24	2.12	6.36
Fold	1.00	1.13	1.30	0.77	0.21	0.39	0.18
CFU/mL (10^2^)	4.90·10^7^	5.20·10^7^	5.40·10^7^	2.15·10^7^	8.00·10^6^	1.60·10^7^	1.20·10^7^
SD	1.41	7.07	4.24	2.12	2.83	2.83	1.41
Fold	1.00	1.06	1.10	0.44	0.16	0.33	0.24
CFU/mL (10^1^)	5.65·10^7^	4.30·10^7^	4.35·10^7^	2.80·10^7^	1.00·10^7^	1.40·10^7^	9.50·10^6^
SD	7.78	4.24	2.12	8.49	4.24	1.41	3.54
Fold	1.00	0.76	0.77	0.50	0.18	0.25	0.17
**MH‐1670**							
CFU/mL (10^4^)	4.00·10^7^	4.05·10^7^	3.75·10^7^	2.35·10^7^	1.25·10^7^	2.10·10^7^	1.70·10^7^
SD	2.83	7.78	0.71	4.95	3.54	5.66	2.83
Fold	1.00	1.01	0.94	0.59	0.31	0.53	0.43
CFU/mL (10^3^)	2.85·10^7^	2.35·10^7^	3.10·10^7^	1.85·10^7^	6.00·10^6^	1.00·10^7^	1.00·10^7^
SD	4.95	9.19	4.24	0.71	4.24	1.41	1.41
Fold	1.00	0.82	1.09	0.65	0.21	0.35	0.35
CFU/mL (10^2^)	2.30·10^7^	2.15·10^7^	1.85·10^7^	1.25·10^7^	5.50·10^6^	1.30·10^7^	4.00·10^6^
SD	2.83	2.12	3.54	2.12	3.54	1.41	1.41
Fold	1.00	0.93	0.80	0.54	0.24	0.57	0.17
CFU/mL (10^1^)	1.00·10^7^	1.20·10^7^	1.60·10^7^	5.00·10^6^	3.50·10^6^	5.50·10^6^	3.50·10^6^
SD	4.24	2.12	1.41	1.41	1.41	2.12	2.12
Fold	1.00	1.20	1.60	0.50	0.35	0.55	0.35
**NCTC 8325**							
CFU/mL (10^3^)	1.65·10^8^	1.34·10^8^	1.45·10^8^	1.12·10^8^	6.55·10^7^	7.00·10^7^	6.85·10^7^
SD	10.21	2.83	2.83	2.83	2.12	1.41	2.12
Fold	1.00	0.81	0.88	0.68	0.40	0.42	0.42
CFU/mL (10^2^)	1.10·10^8^	1.16·10^8^	8.60·10^7^	8.95·10^7^	5.60·10^7^	6.65·10^7^	6.15·10^7^
SD	1.41	4.95	4.24	3.54	2.83	6.36	7.78
Fold	0.67	0.70	0.52	0.54	0.34	0.40	0.37
CFU/mL (10^1^)	1.06·10^8^	8.65·10^7^	7.00·10^7^	5.05·10^7^	4.50·10^7^	5.20·10^7^	4.25·10^7^
SD	6.36	0.71	2.83	4.95	4.24	2.83	0.71
Fold	0.64	0.52	0.42	0.31	0.27	0.32	0.26

**Table A2:** The effect on ATCC 29213 growth after different treatments.

ATCC 29213	Control	IMP‐1700	DMSO	10 Gy + *S. aureus*	10 Gy + IMP‐1700	10 Gy + DMSO
CFU/mL (10^4^)	5.83·10^7^	5.68·10^7^	5.55·10^7^	2.73·10^7^	1.35·10^7^	3.35·10^7^
SD	10.18	7.27	3.51	8.26	4.12	5.07
FOLD	1.00	0.97	0.95	0.47	0.23	0.58
CFU/mL (10^3^)	1.05·10^7^	1.75·10^7^	8.00·10^6^	1.25·10^7^	5.75·10^6^	6.50·10^6^
SD	4.43	2.38	3.56	4.20	1.71	1.73
Fold	1.00	1.67	2.00	1.19	0.55	0.62
CFU/mL (10^2^)	5.75·10^6^	5.25·10^6^	3.00·10^6^	2.00·10^6^	1.75·10^6^	1.00·10^6^
SD	2.22	0.96	1.41	1.41	1.71	1.41
Fold	1.00	0.91	0.52	0.35	0.30	0.26
CFU/mL (10^1^)	1.50·10^6^	1.75·10^6^	2.50·10^5^	2.50·10^5^	5.00·10^5^	1.75·10^6^
SD	1.29	1.50	0.50	0.50	0.58	2.36
Fold	1.00	1.17	0.17	0.17	0.33	1.17

**Table A3:** Bliss independence analysis at fixed doses.

Strain	Treatment	Observed inhibition (%)	Bliss‐predicted inhibition (%)	Interaction
ATCC 29213	CPX (15 µM) + IMP‐1700 (5 µM)	75.7	0.0	Positive
MH‐1670	CPX (15 µM) + IMP‐1700 (5 µM)	68.8	6.3	Positive
NCTC 8325	CPX (15 µM) + IMP‐1700 (5 µM)	59.1	17.0	Positive
ATCC 29213	10 Gy IR + IMP‐1700 (5 µM)	76.8	54.4	Positive

3. **Cell images**

4. **Cell counts**

**Table A4:** The effect on noncancerous cell growth after different treatments.

		NIH‐3T3		
	Hours	24	48	72
Untreated	Mean	1.08·10^5^	3.77E·10^5^	7.36E·10^5^
	Fold	1	1	1
10 Gy	Mean	1.36·10^5^	4.29·10^5^	5.45·10^5^
	Fold	1.26	1.14	0.74
IMP‐1700	Mean	1.11·10^5^	2.54·10^5^	1.43·10^5^
	Fold	1.03	0.68	0.19
10 Gy + IMP‐1700	Mean	9.07·10^4^	1.92·10^5^	1.26·10^5^
	Fold	0.84	0.51	0.17
CPX	Mean	1.67·10^5^	4.71·10^5^	7.81·10^5^
	Fold	1.55	1.25	1.06
10 Gy + CPX	Mean	1.36·10^5^	1.95·10^5^	2.82·10^5^
	Fold	1.26	0.52	0.38
IMP‐1700 + CPX	Mean	1.22·10^5^	2.54·10^4^	2.34·10^5^
	Fold	1.13	0.68	0.32
10 Gy + IMP‐1700 + CPX	Mean	8.35·10^4^	1.25·10^5^	2.34·10^5^
	Fold	0.77	0.33	0.32
DMSO	Mean	1.29·10^5^	3.11·10^5^	7.71·10^5^
	Fold	1.20	0.82	1.05
10 Gy + DMSO	Mean	1.26·10^5^	2.86·10^5^	2.20·10^5^
	Fold	1.16	0.76	0.30

**Table A5:** The effect on tumor cell growth after different treatments.

		B16.F10			MDA‐MB‐231			HepG2		
	Hours	24	48	72	24	48	72	24	48	72
Untreated	Mean	2.33·10^5^	5.90·10^5^	1.82·10^6^	3.21·10^5^	3.95·10^5^	3.99·10^5^	2.89·10^5^	4.69·10^5^	6.45·10^5^
	Fold	1	1	1	1	1	1	1	1	1
10 Gy	Mean	2.47·10^5^	5.87·10^5^	7.47·10^5^	1.48·10^5^	1.59·10^5^	2.55·10^5^	1.09·10^5^	3.75·10^5^	4.52·10^5^
	Fold	1.06	0.99	0.41	0.46	0.40	0.64	0.38	0.80	0.70
IMP‐1700	Mean	2.70·10^5^	3.21·10^5^	2.34·10^5^	9.36·10^4^	4.69·10^4^	6.06·10^4^	1.93·10^5^	1.23·10^5^	1.69·10^5^
	Fold	1.16	0.54	0.13	0.29	0.12	0.15	0.67	0.26	0.26
10 Gy + IMP‐1700	Mean	2.54·10^5^	1.27·10^5^	2.23·10^5^	7.22·10^4^	3.91·10^4^	7.62·10^4^	2.53·10^4^	1.14·10^5^	0.00
	Fold	1.09	0.21	0.12	0.22	0.10	0.19	0.09	0.24	0.00
CPX	Mean	3.09·10^5^	6.81·10^5^	2.60·10^6^	1.76·10^5^	2.41·10^5^	3.74·10^5^	1.76·10^5^	3.19·10^5^	6.20·10^5^
	Fold	1.33	1.15	1.43	0.55	0.61	0.94	0.61	0.68	0.96
10 Gy + CPX	Mean	1.87·10^5^	5.05·10^5^	6.37·10^5^	1.84·10^5^	1.79·10^5^	3.91·10^5^	1.60·10^5^	4.37·10^5^	7.18·10^5^
	Fold	0.81	0.86	0.35	0.57	0.45	0.98	0.55	0.93	1.11
IMP‐1700 + CPX	Mean	2.23·10^5^	2.81·10^5^	2.15·10^5^	1.03·10^5^	1.18·10^5^	7.99·10^4^	1.64·10^5^	1.98·10^5^	2.16·10^5^
	Fold	0.96	0.48	0.12	0.32	0.30	0.20	0.57	0.42	0.34
10 Gy + IMP‐1700 + CPX	Mean	2.85·10^5^	4.03·10^5^	3.36·10^5^	1.68·10^5^	1.10·10^5^	9.38·10^4^	1.80·10^5^	1.48·10^5^	9.77·10^4^
	Fold	1.23	0.68	0.18	0.52	0.28	0.24	0.62	0.32	0.15
DMSO	Mean	1.95·10^5^	7.93·10^5^	1.72·10^6^	2.07·10^5^	2.85·10^5^	5.43·10^5^	4.03·10^5^	7.70·10^5^	4.85·10^5^
	Fold	0.84	1.34	0.95	0.64	0.72	1.36	1.39	1.64	0.75
10 Gy + DMSO	Mean	4.10·10^5^	6.21·10^5^	8.85·10^5^	1.80·10^5^	3.37·10^5^	4.03·10^5^	2.78·10^5^	3.09·10^5^	4.11·10^5^
	Fold	1.76	1.05	0.49	0.56	0.85	1.01	0.96	0.66	0.64
